# Actions of Thyroid Hormones on Thyroid Cancers

**DOI:** 10.3389/fendo.2021.691736

**Published:** 2021-06-21

**Authors:** Shaker A. Mousa, Aleck Hercbergs, Hung-Yun Lin, Kelly A. Keating, Paul J. Davis

**Affiliations:** ^1^ Pharmaceutical Research Institute, Albany College of Pharmacy and Health Sciences, Rensselear, NY, United States; ^2^ Department of Radiation Oncology, The Cleveland Clinic, Cleveland, OH, United States; ^3^ PhD Program for Cancer Molecular Biology and Drug Discovery, College of Medical Science and Technology, Taipei Medical University, Taipei, Taiwan; ^4^ Taipei Cancer Center, Taipei Medical University, Taipei, Taiwan; ^5^ Traditional Herbal Medicine Research Center of Taipei Medical University Hospital, Taipei Medical University, Taipei, Taiwan; ^6^ Department of Medicine, Albany Medical College, Albany, NY, United States

**Keywords:** thyroid hormone, L-thyroxine (T4), integrin αvβ3, thyrotropin (TSH), tetraiodothyroacetic acid (tetrac), follicular thyroid carcinoma, differentiated thyroid cancers

## Abstract

L-Thyroxine (T4) is the principal ligand of the thyroid hormone analogue receptor on the extracellular domain of integrin αvβ3. The integrin is overexpressed and activated in cancer cells, rapidly dividing endothelial cells, and platelets. The biologic result is that T4 at physiological concentration and without conversion to 3,3’,5-triiodo-L-thyronine (T3) may stimulate cancer cell proliferation and cancer-relevant angiogenesis and platelet coagulation. Pro-thrombotic activity of T4 on platelets is postulated to support cancer-linked blood clotting and to contribute to tumor cell metastasis. We examine some of these findings as they may relate to cancers of the thyroid. Differentiated thyroid cancer cells respond to physiological levels of T4 with increased proliferation. Thus, the possibility exists that in patients with differentiated thyroid carcinomas in whom T4 administration and consequent endogenous thyrotropin suppression have failed to arrest the disease, T4 treatment may be stimulating tumor cell proliferation. *In vitro* studies have shown that tetraiodothyroacetic acid (tetrac), a derivative of T4, acts *via* the integrin to block T4 support of thyroid cancer and other solid tumor cells. Actions of T4 and tetrac or chemically modified tetrac modulate gene expression in thyroid cancer cells. T4 induces radioresistance *via* induction of a conformational change in the integrin in various cancer cells, although not yet established in thyroid cancer cells. The thyroid hormone receptor on integrin αvβ3 mediates a number of actions of T4 on differentiated thyroid cancer cells that support the biology of the cancer. Additional studies are required to determine whether T4 acts on thyroid cancer cells.

## Introduction

The extracellular domain of plasma membrane integrin αvβ3 contains a receptor for thyroid hormone analogues (1). The functions of this site on the integrin have been examined in several reviews (2, 3). Integrins are transmembrane dimer proteins that are important to cell-cell interactions and to plasma membrane interactions with extracellular matrix proteins (4). L-thyroxine (T4) is, at physiological concentrations, the principal ligand of the cell surface iodothyronine receptor on αvβ3, rather than serving in the role of a prohormone for 3,3’,5-triiodo-L-thyronine (T3) that acts intracellularly at nuclear receptors and on mitochondria (3, 5).

Integrin αvβ3 with its T4 receptor on the integrin is overexpressed by cancer cells and frequently is also activated in tumor cells, assuming a configuration that fosters cell-cell interaction and binding of extracellular proteins (4). We have shown that activation of αvβ3, i.e., an extended conformation, is regulated by radiation and thyroid hormone analogues (6). Overexpression and activation of the protein renders tumor cells susceptible to actions of T4 to stimulate proliferation, to support cancer cell defense pathways such as anti-apoptosis (7) and radioresistance (8), and to enhance cancer-related angiogenesis (2, 3, 9, 10). Preclinical studies have shown a variety of tumor cells to proliferate in response to physiological concentrations of T4 (11–14). A derivative of T4, tetraiodothyroacetic acid (tetrac), blocks actions of T4 on tumor cell αvβ3 (3). Preliminary clinical evidence indicates that lowering of systemic T4 levels and maintaining normal metabolic state with T3 (euthyroid hypothyroxinemia) (15) slows growth of multiple types of solid tumors. Clinical hypothyroidism has been shown in multiple reports specifically to slow the clinical course of renal cell carcinoma (16), breast cancer (17), glioblastoma (18), and head-and-neck cancers (19). Such evidence confirms that T3 has little activity at the integrin receptor for thyroid hormone analogues.

Among the solid tumor cells whose proliferation *in vitro* have been shown to be stimulated by T4 are thyroid cancers, such as differentiated follicular thyroid carcinoma (20). Such tumors are usually thyrotropin (TSH)-dependent and managed clinically with exogenous T4 in modest excess to suppress host pituitary TSH production. The recognition of the thyroid hormone receptor for T4 on integrin αvβ3 and its contribution to differentiated thyroid cancer cell proliferation raises the possibility that when TSH suppression is sometimes ineffective in thyroid tumor management, the tumor is manifesting T4-dependence, rather than TSH-dependence, as we have pointed out elsewhere (21). T4 can convey radioresistance to tumors *via* αvβ3 (6, 8). A question may then arise in thyroid cancer management when radiation therapy by several mechanisms has been ineffective, whether endogenous T4 or TSH-suppressive T4 is contributing to the reduced effectiveness of the radiation. This review examines these treatment scenarios.

## Control of Growth of Differentiated Thyroid Cancers and Thyroid Hormones

Pituitary TSH is a principal host growth factor for differentiated papillary and follicular thyroid cancers. Suppression of this factor *via* negative feedback inhibition with exogenous L-thyroxine is a routine component of management of these tumors. The TSH acts at a specific cell surface G protein-coupled receptor (GPCR) in differentiated thyroid carcinomas. The discovery of the cell surface receptor for thyroid hormone analogues on the extracellular domain of integrin αvβ3 that is overexpressed by cancer cells and active endothelial cells raised the possibility that thyroid hormones as T4 might be, like TSH, a circulating growth factor for solid tumors, including thyroid cancers. In such a clinical setting, the relative importance of the TSH receptor and the integrin thyroid hormone analogue receptor can be an issue. In practical terms, however, the management strategy of T4 administration in patients with differentiated thyroid cancers is usually effective in arresting tumor growth; this indicates that the TSH receptor is of greater importance than the integrin receptor for T4 in most patients. We have pointed out, however, that when suppression of endogenous TSH with T4 is ineffective and thyroid cancer grows appreciably, the tumor is likely to be T4- and integrin-dependent (21).

As mentioned above, the integrin-dependent, trophic effect of T4 on cancers of various tissues is complex and involves increased expression of genes relevant to cell division, anti-apoptosis, tumor-relevant angiogenesis, and radioresistance (2, 3). Tetrac and chemically modified tetrac arrest the growth of two xenograft models of human differentiated follicular thyroid carcinoma (22). A component of this response was shown to be anti-angiogenesis. These results indicate that the integrin αvβ3 thyroid hormone receptor contributes to regulation of tumor growth. We have shown that both follicular thyroid carcinoma and papillary thyroid carcinoma cells proliferate *in vitro* with exposure to physiological concentrations of T4 and that this response is dependent upon activation of mitogen-activated protein kinase MAPK (23). The tetrac effects and involvement of MAPK in these actions of T4 on thyroid cancers indicate involvement of a direct, nongenomic action of T4 on the tumor cells. Thus, in clinical settings when endogenous TSH is fully suppressed by administration of T4, but differentiated thyroid cancer cells continue to proliferate, the proliferation may reflect actions of T4 at the thyroid hormone receptor on the tumor cell surface integrin (21). A wholly different form of thyroid cancer, medullary carcinoma of the thyroid gland, has also been shown to have T4-dependent proliferation (24).

Whether T4 may also render poorly differentiated thyroid cancers more aggressive has not as yet been determined. T3 is not an active ligand of the thyroid hormone receptor on integrin αvβ3 and there is clinical evidence that T3 instead of T4 may be safely administered to patients with a variety of cancers, for example, with systemic methimazole inhibition of thyroid gland synthesis of iodothyronines (15).

## Gene Expression in T4-Treated Thyroid Cancer Cells

Modulation in various types of tumor cells—e.g., glioblastoma, pancreatic cancer, kidney—of the expression of specific genes by hormone ligands at the thyroid hormone receptor on integrin αvβ3 has been recently reviewed (2, 23). Of particular importance are actions of T4 to enhance signal transduction in papillary and follicular thyroid cancer cells (MAPK/ERK pathway) and on apoptosis-relevant genes (23). T4 prevents thyroid cancer cell accumulation of pro-apoptotic p21, c-Fos, and c-Jun proteins by blocking induction of expression of the genes for these moieties. Additional information is needed about the effects of T4 and other hormone analogues on gene expression in thyroid cancers.

## Thyrotropin (TSH) Receptor and Integrin αvβ3

The TSH receptor (TSHR) is a trans-membrane GPCR that is internalized periodically (25) and may move to the trans-Golgi network (TGN) in thyroid cells. This can lead to downstream protein kinase A (PKA) signaling to the nucleus (26). Integrin trafficking is also an extensive process (27), and we have shown that T4 stimulates tumor cell internalization of αvβ3 (28) and function of the αv monomer as a coactivator protein in the nucleus. T4 can activate PKA (29, 30). Various GPCRs are known to interact with integrins (31), and extracellular matrix proteins such as vitronectin or fibronectin have been shown to bind to αvβ3 (3) and with TSHR (32), altering functions of the proteins or promoting internalization. This body of evidence has not, however, been examined in thyroid cancer cells for the possibility that TSHR and αvβ3 traffick together in these cells when T4 binds to the integrin. Were such coupling to occur, then circulating levels of TSH would have reduced cancer cell target TSHRs to which to bind and act. One additional note of possible relevance is that, under certain circumstances, TSH may modulate abundance of αvβ3 in specific tissues, such as endothelium (33). It would appear worthwhile for the trafficking of the integrin and TSHR to be studied concurrently in thyroid cancer cells in the presence and absence of T4.

A recent review has concluded that TSH suppression in patients with intermediate-risk or high- risk differentiated thyroid cancers may be of limited benefit (34). It will be of interest and importance to determine whether this is a function of increased susceptibility of such tumors to T4-induction of radioresistance *via* integrin αvβ3.

## Tumor Cell Respiration/ATP Production and the Thyroid Hormone Analogue Receptor on Integrin αvβ3

We have recently reported that a large number of tumor cell respiration-linked genes are regulated from the integrin by thyroid hormone analogues. This study depended upon a tetrac-containing agent to probe the function of thyroid hormone analogue receptor function. Among the genes affected were multiple ATP synthases and NADH dehydrogenases (35). Prior to this study, the control of cell respiration—in normal cells, as well as tumor cells—was thought to be an exclusive function of T3 at the level of mitochondria and of ATP production-related genes in the nucleus of cells. An implication of these tetrac-based observations is that T4 may regulate respiration in cancer cells from the cell surface integrin. This possibility has not yet been explored.

## Metastasis of Thyroid Carcinomas and Possible Roles of T4

We have indicated elsewhere that certain actions of T4 on tumor cells and on platelets may support the process of metastasis from primary cancers (36). These actions of T4 include cell proliferation, cell migration, platelet-tumor cell interaction, and angiogenesis (36, 37). The T4 antagonist, tetrac, acts at αvβ3 to inhibit the metastatic process in a breast cancer model (36). Such inhibition needs to be examined in a thyroid cancer metastasis model (38).

## Possible Actions of Thyroid Hormone Analogues Other Than T4 and Tetrac on Thyroid Cancer Biology

In the context of thyroid hormone actions in normal, nonmalignant cells, T3 is the active form of the hormone (3, 5, 39). We have shown elsewhere and emphasized here that in cancer cells and dividing endothelial cells, however, T4 and tetrac are the active forms of the hormone. We would note that reverse T3 (rT3) also appears in certain tumor cells to have proliferative activity at the integrin (40, 41), but not at nuclear receptors. rT3 activity has not yet been examined for activity in thyroid cancer cells.

## Immune Checkpoint PD-L1/PD-1 and Thyroid Carcinoma

The programmed cell death protein 1 (PD-1) and the programmed death ligand 1 (PD-L1) are an immune checkpoint that has been well-studied in different types of thyroid carcinomas (42–44). Tumor production of PD-L1 is a marker of aggressiveness. We know from studies in a variety of solid tumors other than thyroid cancer that express PD-L1 that the thyroid hormone analogue receptor on integrin αvβ3 regulates release of PD-L1 (45). Tetrac and chemically modified tetrac will prevent tumor cell production/release of PD-L1. It is thus of some importance for the behavior of PD-L1 production to be studied as a function of ambient T4 in thyroid carcinomas. That is, the use of T4 to suppress endogenous TSH in patients with aggressive differentiated thyroid cancers may undesirably support PD-L1 production by the tumor and block any host anti-tumor immune response that is present.

## Thyroid Hormones and Radioresistance in Cancer Cells

Leith and co-workers (8, 46) have reported that the thyroid hormone analogue receptor on integrin αvβ3 regulates the state of radioresistance of certain cancer cells. This appears to be a function of hormone-linked changes in the conformation of the integrin (6) that require hormone-binding to αvβ3. These observations raise the possibility that host thyroid gland-produced T4 or exogenous T4 intended to suppress endogenous TSH may lessen the impact of therapeutic radiation on the thyroid tumor. However, the T4-radioresistance studies have been conducted in tumor cells other than thyroid carcinoma and require repetition in thyroid cancer cells.

Various factors that appear to contribute to radioresistance of thyroid cancers have recently been summarized in a radioiodine refractoriness score (47). The factors in this score include history of cigarette smoking and local tumor extension. We suggest that circulating free T4 quartile levels be considered, together with other measures (47, 48) for such evaluation.

## Tetrac as a Pharmacological Inhibitor of Integrin-Dependent Actions of T4

It has been noted in several sections of this review that the T4 analogue, tetrac, or chemical modifications of tetrac, can block trophic actions of T4 on thyroid cancer cells. Several chemical modifications of tetrac have been developed and tested preclinically as prospective anticancer agents (14, 49, 50). Tetrac also has intrinsic anticancer activity in the absence of T4 (3, 5).

## Discussion

We have emphasized in this review the contribution of the thyroid hormone analogue receptor on integrin αvβ3 to the behaviors of thyroid cancer cells. From the standpoint of tumor cell biology, T4 is the principally active iodothyronine at this plasma membrane hormone receptor site in differentiated thyroid cancer cells. T3 in physiological concentrations has little or no bioactivity at this receptor. Differentiated human follicular and papillary thyroid carcinoma cells proliferate in response to T4, but poorly differentiated/anaplastic thyroid cancer cells have not been specifically examined for responses to T4. Medullary carcinoma of the thyroid gland is responsive to T4 (24). The proliferative activity of T4 is opposed by tetrac or chemically modified tetrac in the thyroid carcinoma cells in which actions of T4 have been examined.

We emphasize here that the actions of thyroid hormone analogues on thyroid cancers need to be explored more fully. TSH is a critical growth factor for the majority of thyroid cancers. But if T4 is a trophic factor for thyroid cancers and is being employed clinically as a suppressor of host TSH, we need to know whether the tumor cells in patients who have not responded to TSH suppression are now T4-dependent (21). This can be determined by imposition of the state of euthyroid hypothyroxinemia (15). Metastasis of thyroid cancer may also have some dependence on availability of T4 to thyroid tumor cells.

PD-L1 is an important product of certain thyroid cancer cells (42–44). We know from studies in a variety of nonthyroid cancer cells that T4 enhances the production of this immune checkpoint component that blocks immune attacks on cancer cells. When thyroid cancers progress in the context of satisfactory suppression of host TSH, we may also ask whether immune checkpoint actions of T4 in the TSH-suppression treatment approach is blocking any immune system activity that might be present and potentially relevant to clinical management.

We have raised the possibility elsewhere that T4 may contribute to the metastatic activity of cancers (36). In distant metastases of various types of cancer, the interaction of platelets and tumor cells that is fostered by T4 may contribute to metastasis formation (37). It is not clear what the factors are that lead to local—particularly, central—lymph node metastases from differentiated thyroid cancers (51). We propose that local T4 production by thyroid cancer cells may be a factor that contributes to local, as well as distant, metastasis. The actions of T4 at tumor sites will also foster angiogenesis that is cancer- relevant (9, 10).

There is a substantial literature on specific microRNA (miRNA) elaboration by thyroid cancers (52–54). Abundance of these moieties in thyroid tumors may correlate with tumor type and size, metastasis, and recurrence. The possible influence of T4 on specific miRNA production in thyroid tumors has not yet been determined.

Other molecular mechanisms may be involved in the genesis of thyroid cancers (55, 56), such as a multicomponent signaling system involving fibroblast growth factor receptor, mutated BRAF signaling, and other communication pathways (57). It may be useful to examine such a mechanism for participation by T4 because of its nongenomic actions on a variety of signal transduction pathways (3).

A panel of interleukins may contribute to regulation of thyroid cancer cell proliferation and migration (58). Acting at the receptor on integrin αvβ3, thyroid hormone analogues regulate the inflammatory response *via* various interleukins and chemokines (59). Aside from relevance to neoplasia, cytokine storms potentiated by T4 have been proposed to support infection-related inflammatory states (60).

Finally, T4 may support radioresistance in a variety of tumor cells, but T4 has not yet been examined for such an effect in thyroid cancers. It is routine to withhold TSH-suppressive T4 administration in thyroid cancer patients until ablative radioactive iodine has been administered. An effect of withholding T4 should be a change in conformation of integrin αvβ3 to the non-radioresistant configuration (6). The extent of conformation change of integrin αvβ3 can be readily examined immunologically and graded in thyroid tumor biopsy prior to radio-ablation. When the extended conformation is in excess in cancer cells, a formula might be developed for consideration of brief administration of a tetrac-containing agent before radiation treatment. The molecular basis of tetrac-induced radiosensitivity involves multiple signal transduction pathways in the tumor cells (8).

## Author Contributions

PD reviewed the literature and wrote the paper. All authors contributed to the article and approved the submitted version.

## Conflict of Interest

SM and PD are the founders of NanoPharmaceuticals LLC, that is developing chemically modified forms of tetrac for application to management of various types of cancer, and PD is Chief Scientific Officer at the company.

The remaining authors declare that the research was conducted in the absence of any commercial or financial relationships that could be construed as a potential conflict of interest.
